# Multiparametric Optimization of Data-Dependent Acquisition Towards More Holistic Bacterial Metabolite Coverage Through Molecular Networking

**DOI:** 10.1155/ijm/4388417

**Published:** 2025-07-21

**Authors:** Adivhaho Khwathisi, Amidou Samie, Asfatou Ndama Traore, Ntakadzeni Edwin Madala

**Affiliations:** Department of Biochemistry and Microbiology, University of Venda, Thohoyandou, South Africa

**Keywords:** bacterial metabolites, data-dependent acquisition (DDA), LC-MS (liquid chromatography-mass spectrometry), metabolite profiling, metabolomics, molecular networking

## Abstract

Prokaryotic organisms rely on a limited array of metabolites for survival, which varies according to their natural environment. For example, soil-borne bacteria produce diverse metabolites, such as antibiotics, to thrive in their competitive surroundings, inhibiting the growth of nearby competing bacteria. The structural diversity of these compounds offers great analytical challenges, since there is no universal acquisition setting that can be applied to achieve their comprehensive coverage. Therefore, the use of a single experimental setup inevitably hinders the comprehensive metabolite coverage, which would affect the outputs. To address this, we propose employing a design of experiment (DoE) approach through the central composite design (CCD) to enhance the metabolite detection and broaden the coverage of the data-dependent acquisition (DDA) mode of the UHPLC-qTOF-MS technique. Our study reveals that altering collision energy significantly enhances metabolite coverage compared to adjusting the DDA threshold of detection. Furthermore, the ability of global natural product social (GNPS)–based molecular network models to annotate metabolites is greatly influenced by data acquisition settings, particularly affecting MS^2^ data. Interestingly, molecular networks constructed from averaged spectral data obtained through randomly selected DDA settings outperform those generated using customized settings through DoE modeling. This study demonstrates that in untargeted LC-MS metabolomics, both collision energy and intensity threshold independently enhance metabolite coverage in untargeted metabolomics. However, their combined use results in even greater coverage. Consequently, we recommend adopting group-based optimization over single-point optimization for more comprehensive metabolite coverage and in-depth exploration. However, caution should be taken in order to balance between robust data and redundancy.

## 1. Introduction

Microorganisms, particularly bacteria, play crucial roles in the natural environment through various interactions with plants. These interactions can be mutualistic, such as nitrogen fixation [1], or parasitic, affecting plant health [2]. The interactions are facilitated by environmental sensing and the exchange of signals into molecular and genetic information [3, 4]. However, to ensure their survival and adaptation to their environment, microorganisms must produce a diverse range of secondary metabolites, which significantly impact their interaction with both biotic and abiotic factors [5]. Key mechanisms include the exchange of secondary metabolites [6], production of antimicrobial compounds [7], signaling molecules [8], and genetic elements [9], influencing microbial colonization and establishment [10]. These bacterial metabolites serve as antibiotics, pigments, growth hormones, and antitumor agents, highlighting their significance in maintaining human health and well-being [11].

Given that these compounds are not essential for the microorganism's growth and development and their production is regulated by cryptic gene clusters, which are inactive under normal conditions, their comprehensive analysis is challenging [12]. Previous studies have shown that the number of metabolites in bacteria can be affected by various factors, including the type of bacteria, the growth conditions, and the analytical methods used for detection [13]. However, the use of advance analytical techniques such as liquid chromatography–mass spectrometry (LC-MS) in biological systems has emerged as an effective technique for analyzing metabolites due to its capability to detect and characterize compounds with diverse structural differences [14–16]. Additionally, these instruments generate large datasets, and handling and analyzing such large datasets can be challenging and may require specialized data analysis strategies and computational resources [17].

Recently, efforts have been centered on tackling the challenge of mining mass spectral data, which has emerged as a key focus in initiatives aimed at improving metabolite profiling [18]. These recent approaches usually define the substructure and their relationship to molecular fingerprints, annotating chemical compound classes and creating mass spectral networks from metabolomics data [19]. Molecular networking, housed in the global natural product social (GNPS) science is an effective tool for analyzing and visualizing mass spectral data [17, 20]. This approach involves grouping compounds based on their spectral similarity, allowing for the visualization of chemical structural relationships between detected metabolites solely through their MS^2^ spectral data [17, 21]. Previously, studies have used molecular networking to identify and explore the structural relationship between various compounds, aiding the discovery of novel metabolites and their potential application in medical fields [16, 22]. This technique provides insight into the diverse chemical profiles of bacterial populations and facilitates the identification of unique bioactive compounds [23]. However, the molecular networking approach heavily relies on the MS/MS spectra generated to deduce spectral similarities, which implies that the quality of MS^2^ data is fundamentally essential for reducing redundancy in generating informative molecular networks [24].

Over the last decade, the use of advanced MS data acquisition approaches such as data-dependent acquisition (DDA) [25] and data-independent acquisition (DIA) approaches has been increasingly used to provide both high resolution and high throughput for a full metabolome composition of the organisms [26]. DDA involves selecting specific ions for fragmentation based on their intensity or abundance, while DIA involves fragmenting all ions within a predetermined mass-to-charge ratio (*m*/*z*) range, regardless of their intensity or abundance. However, DDA is a widely used technique and preferred for its high spectral quality and time efficiency, especially for metabolite profiling and annotation in untargeted metabolomics. Despite its limitations in capturing the complete spectrum of metabolites (metabolome), [25] introduced eight guidelines for selecting technical parameters to enhance the effectiveness of DDA analyses. Given all these improvements, the chemical heterogeneity of metabolites poses a significant challenge in achieving holistic metabolite coverage even with optimal LC-MS conditions, which therefore requires the continuous development of new technologies and standards to expand the coverage of metabolites and improve the quality and robustness of the data. However, efforts to optimize the DDA parameters have been done through UHPLC-qTOF-MS analyses of plant extracts [24] and orbitrap-based mass spectrometers [27] for plant compounds, but none has been done for bacterial metabolites. Herein, we propose employing a design of experiment (DoE) approach through the central composite design (CCD) to enhance the metabolite detection and broaden the coverage of the DDA mode within the UHPLC-qTOF-MS technique.

## 2. Experimental

### 2.1. Metabolite Extraction

Pure bacterial isolates were cultured in Mueller Hinton Broth for 24 h to facilitate bacterial cell harvesting. Each Erlenmeyer flask containing 200 mL of nutrient broth received an inoculation of 1 mL from the bacterial stock solutions. Following incubation, bacterial cells were harvested by centrifugation at 5000 rpm for 15 min at 4°C. The yielded supernatants were subsequently transferred into new 50-mL conical tubes. The supernatants were then filtered through 0.22-*μ*m nylon filters into LC-MS glass vials, following established protocols [28]. Furthermore, sample preparation involved three biological replicates, each analyzed in three technical replicates to ensure reproducibility.

### 2.2. LC-MS Analysis

The analysis of bacterial methanolic extracts was conducted using a Shimadzu LC-q-TOF-MS, a 9030 model equipped with a Shim-pack Velox C18 column (100 × 2.1 mm, particle size 2.7 *μ*m), as previously described by [29]. The column temperature was maintained at 50°C throughout the analysis. A 5-*μ*L injection volume was used, and samples were separated over a 30-min binary gradient at a constant flow rate of 0.3 mL/min. The gradient employed a solvent mixture of water with 0.1% formic acid (Eluent A) and methanol with 0.1% formic acid (Eluent B). Specifically, the gradient elution was as follows: Eluent B was maintained at 5% from 0 to 3 min, increased linearly from 5% to 40% between 3 and 8 min, further increased to 95% between 8 and 23 min, held isocratically at 95% from 23 to 25 min, returned to 5% from 25 to 27 min, and re-equilibrated at 5% from 27 to 30 min. The LC eluents were then introduced into a quadrupole time-of-flight high-definition mass spectrometer operating in positive electrospray ionization (ESI) mode. The q-TOF-MS conditions included a 400°C heat block temperature, 250°C desolvation line temperature, 42°C flight tube temperature, and a nebulization and dry gas flow rate of 3 L/min. Data acquisition was performed in DDA mode, simultaneously capturing both MS^1^ and MS^2^ spectra for ions within a *m*/*z* range of 100–2000 and an intensity threshold exceeding 1000. During MS^2^ experiments, collision-induced dissociation employed argon gas with a collision energy of 30 eV and a spread of 5, utilizing sodium iodide (NaI) as a calibration solution to ensure high mass precision. For enhanced coverage, DDA conditions were also intentionally altered (Table 1).

### 2.3. Metabolite Annotation and Molecular Networking

Molecular networks were generated using GNPS molecular networking platform (http://gnps.ucsd.edu). Prior to uploading to the online workflow, UHPLC-qTOF-MS/MS raw data was converted to mzML format. Upon uploading spectral data, MS^2^ fragment ions within +/− 17 Da of the precursor *m*/*z* were filtered out. The minimum matched fragments between consensus spectra to be connected by an edge were set as 5. An MS-CLUSTER technique was used to cluster the data. The MS^2^ fragment ion and a mass tolerance for the precursor ion were both set at 0.02 Da. Edges (representing interconnections/similarities between metabolites) were formed only when more than six matching peaks were present, and a minimum cosine score of 0.7 was exceeded. In the network, only nodes that ranked within each other's Top 10 similar nodes (TopK value of 10) were retained, with a maximum of 100 nodes per molecular family. All matched and some mismatched nodes were annotated based on empirical formulas derived from accurate mass and fragmentation patterns obtained from MS^2^ studies. These annotated metabolites were further validated against literature and searched against common dereplication databases for natural products (e.g., KNApSAcK). Metabolite annotation adhered to the Metabolomics Standards Initiative (MSI) Level 2 guidelines [30]. Classical molecular networking and Spec2vec methodologies were employed to assess the number of nodes, library IDs, and self-loops within the networks. The generated networks were visualized using Cytoscape 3.10.1.

### 2.4. Statistical Analysis

The central composite design response surface methodology (CCD RSM) was employed for experimental design and analysis to establish the relationship between factors, aiming to optimize metabolite coverage using Design-Expert. A two-level full factorial CCD was implemented, comprising a total of 10 experimental runs (including two repetitions). Model parameters and significance were assessed at *p* < 0.005, with the model's adequacy evaluated based on the coefficient of regression (*R*^2^) obtained from the analysis of variance (ANOVA). The model fit produced a response surface, depicting the behavior of the response variable. These plots facilitated the identification of optimized factor ranges that maximize the desired response. Upset plots and Venn diagrams were created using the online platform (https://www.bioinformatics.com.cn) for data analysis and visualization.

## 3. Results

**Figure 1:**
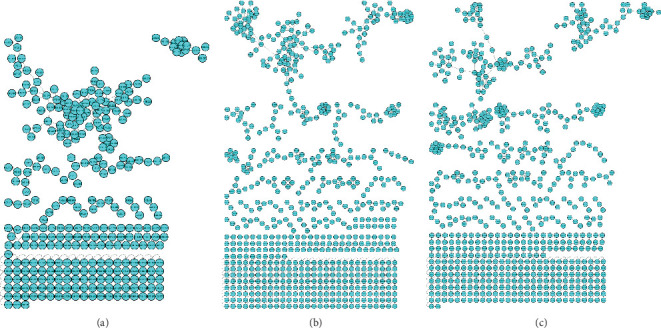
Molecular networks derived from bacterial extracts illustrating the effect of collision energy on MS data acquisition. (a) Collision energy set at 10 eV with a 1000 threshold. (b) Collision energy set at 20 eV with a 1000 threshold. (c) Collision energy set at 30 eV with a 1000 threshold.

Bacterial extracts were analyzed using LC-MS operating in DDA mode. The generated data were processed using molecular networking and visualized with Cytoscape. In the current study, the effect of collision energy was assessed to examine its effect on metabolite coverage when the threshold of intensity was kept constant. Using GNPS-based molecular networking, the MS/MS data were organized into nodes and clusters, with the nodes indicating the number of metabolites and clusters representing molecular families of metabolites/compounds that showed spectral similarities [17]. Herein, the number of nodes showed an increase with the increase of the collision energy, with 10 eV exhibiting the fewest metabolites and 30 eV displaying the highest when the threshold of intensity was kept at 1000, as depicted in Figure 1.

**Figure 2:**
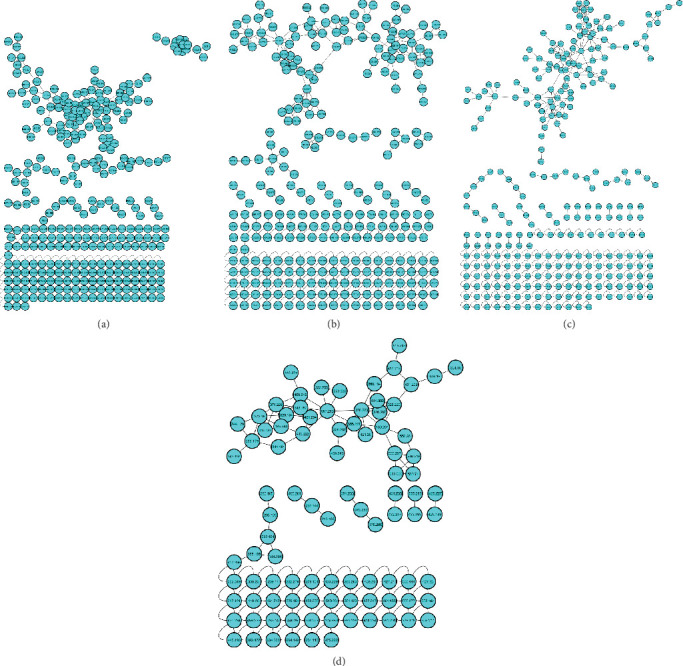
Molecular networks derived from bacterial extracts illustrating the effect of threshold intensity on MS data acquisition. (a) 1000 threshold with collision energy set at 10 eV. (b) 2000 threshold with collision energy set at 10 eV. (c) 3000 threshold with collision energy set at 10 eV. (d) 4000 threshold with collision energy set at 10 eV.

To further explore the impact of the threshold of intensity on metabolite coverage, molecular networks were constructed using the MS/MS data when the collision energy was kept constant at 10 eV. The influence of the threshold of intensity was distinctly evident in the generated molecular networks (Figure 2). Lowering the threshold allowed more ions to be detected, resulting in a broader coverage of metabolites within the network. Specifically, Figure 2d, where the threshold of intensity was set at 4000, detected the fewest number of metabolites. In contrast, Figure 2b, with a threshold of intensity set at 2000, detected the highest number of metabolites. This demonstrates that a lower intensity threshold enhances the detection sensitivity, leading to a more comprehensive identification of metabolites in the analysis.

**Figure 3:**
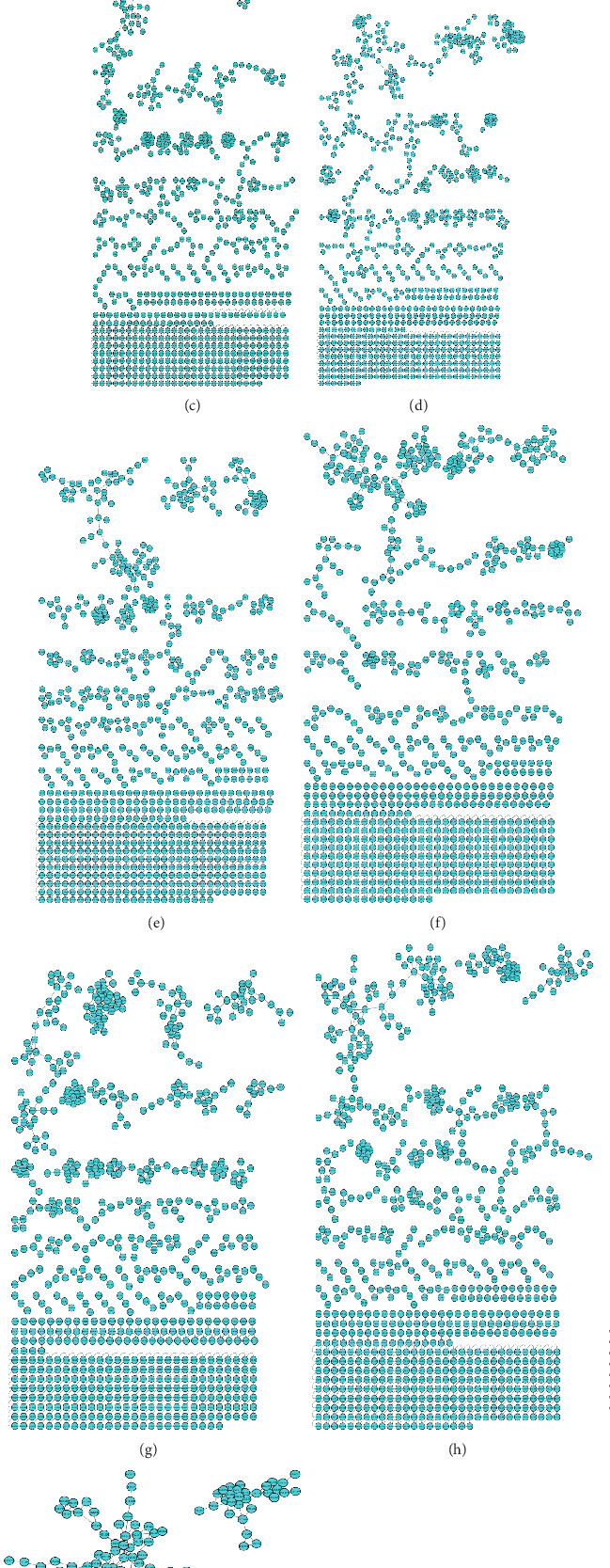
Showing the different molecular networks generated from the design of experiment (DoE) analysis of threshold and collision energy, visualized in Cytoscape. (a) Threshold of 2000 with 20 eV. (b) Threshold of 3000 with 10 eV. (c) Threshold of 1000 with 30 eV. (d) Threshold of 3000 with 30 eV. (e) Threshold of 2000 with 20 eV. (f) Threshold of 585 with 20 eV. (g) Threshold of 2000 with 34 eV. (h) Threshold of 3414 with 20 eV. (i) Threshold of 1000 with 10 eV. (j) Threshold of 2000 with 5 eV.

Additionally, to enable the identification of optimal conditions that would improve metabolite coverage, we opted to investigate the two factors (collision energy and threshold of intensity) simultaneously using a DoE approach. Subsequently, the models generated were used to acquire MS data, which was then used to construct molecular networks for analysis and visualization.  Figure 3 shows the different molecular networks generated from the DoE analysis of threshold and collision energy, visualized in Cytoscape: (A) threshold of 2000 with 20 eV, (B) threshold of 3000 with 10 eV, (C) threshold of 1000 with 30 eV, (D) threshold of 3000 with 30 eV, and (E) threshold of 2000 with 20 eV. Evidently, among the various metabolites detected in each condition, there exists a subset of shared metabolites, suggesting a common pool of metabolites detected across all conditions. These shared metabolites may possess structural features that enable their detectability regardless of experimental conditions. In addition, the effect of collision energy on the data is evident; as the collision energy increased, there was a corresponding rise in the number of detected metabolites, attributed to the increase in fragment ions (Figure S1). Furthermore, the impact of the threshold of intensity was also observed, such that decreasing the threshold of intensity resulted in a greater number of metabolites being detected (Figure 3f).

**Figure 4:**
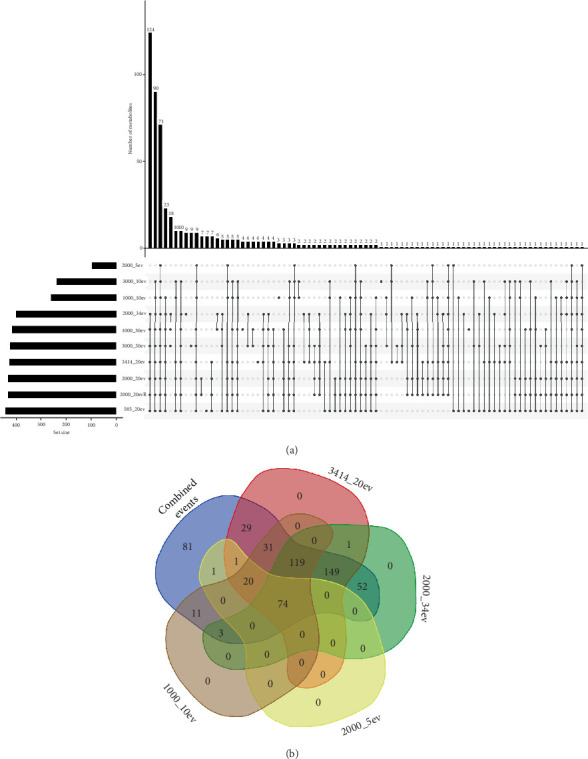
(a) Upset plot illustrating the overlap of different metabolites among the 10 experimental conditions. Each circle represents a set of metabolites associated with a specific condition, with the size of the circle indicating the number of metabolites in that set. The bars along the top of the plot show the number of metabolites unique to each condition, while the intersections between circles represent the overlap of metabolites between conditions. (b) Venn diagram showed the overlapped metabolites between four different conditions with the combined events.

To have an in-depth understanding of the synergistic effect of collision energy and threshold of intensity on metabolite coverage, the data derived from the molecular network was utilized to visually represent the distribution of metabolites captured using various conditions through an upset plot (Figure 4). The results showed that the shared metabolites across all conditions were 71, which approximately accounted for 13.23%, while unique metabolites specific to individual conditions ranged from 0.18% to 1.67%. In addition, combining the spectral data in the GNPS platform results in an increased coverage of the detected metabolites. This observation aligns with the findings depicted in the Venn diagram in Figure 4, highlighting the interactions between the highest, lowest, and combined events. Similarly, when all conditions were combined, there was a significant increase in metabolite coverage. This suggests that while collision energy and threshold of intensity independently enhance metabolite coverage, the coverage is even greater when both parameters are combined. The results highlight the importance of optimizing both collision energy and threshold of intensity for comprehensive metabolite coverage.

**Figure 5:**
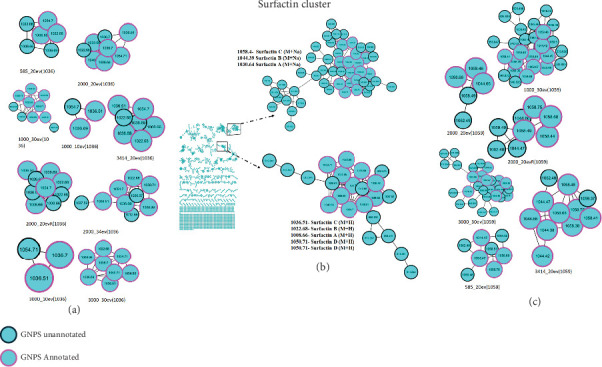
Molecular networking analysis of the methanolic extracts of *B. subtilis* generated from various conditions. (a) The molecular network shows distinct clusters of surfactin at varying collision energies and thresholds detected at M + H. (b) Improved clustering of surfactin is depicted when combining different conditions. (c) The clustering of surfactin detected in M + Na is influenced by varying collision energy and ion detection threshold across different conditions.

As mentioned, the present study is aimed at maximizing the coverage of metabolites from *Bacillus subtilis* by quantitatively and systematically assessing the effects of collision energy and threshold of intensity. The analysis revealed that *Bacillus subtilis* contained a diverse array of metabolite classes, including dipeptides, lipopeptides, and organic compounds. The annotation of the detected metabolites was facilitated by matching the data against the GNPS library. Notably, the annotation of the metabolites showed a marked increase when the data from all the experimental conditions were combined. While there were significant improvements in metabolite annotation observed with individual condition optimizations, such as increasing the collision energy and decreasing the threshold of intensity, the greatest improvement in annotation was achieved when the heterogeneous data from all the conditions were combined. This suggests that leveraging the complementary information captured across the varied experimental settings led to a more comprehensive metabolite coverage and identification. Furthermore, a cluster of biologically significant compounds (surfactins) were used as a model to analyze the effect of threshold and collision energy simultaneously. As observed in Figure 5, the collision energy and threshold of intensity were found to have a significant effect on the detection of metabolites. Precisely, lower collision energies and higher thresholds of intensity led to fewer compounds being detected, whereas higher collision energies and lower thresholds of intensity resulted in a greater number of compounds being detected. Additionally, some compounds were observed to form sodium (Na) adducts. Notably, these compounds with different adducts were clustered in distinct groups within the molecular network, with a greater number of metabolites in the M + Na group compared to the M + H group.

**Figure 6:**
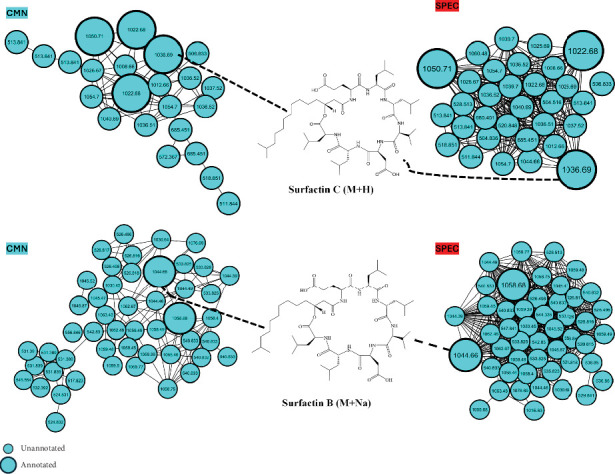
Displays the classical molecular network and Spec2vec networks generated from ESI-positive MS/MS spectra, utilizing consolidated data settings to highlight variations in the clustering of related ion species across different methods. On the left side, the surfactin-extracted cluster is shown from classical molecular networking, while the right side depicts the surfactin-extracted cluster from Spec2vec. This comparison underscores the differences in how these methodologies organize and present molecular relationships within the *Bacillus subtilis* extracts.

**Figure 7:**
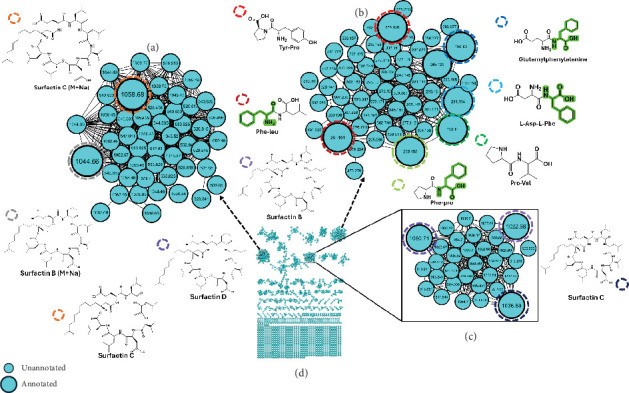
The molecular network of *Bacillus subtilis* extracts was analyzed using liquid chromatography-tandem mass spectrometry with electrospray ionization in positive mode. In the network, (a) surfactins with M + Na adduct, (b) dipeptides, (c) surfactin with M + H adduct, and (d) full molecular network. Each node represents a compound detected in the samples, with edges connecting nodes indicating spectral similarity between the compounds.

The findings from classical molecular networking showed that most clustered nodes were distributed across all DDA settings. However, a significant proportion of these nodes appeared to originate exclusively from settings characterized by high collision energy and low intensity of thresholds. To complement these observations, the Spec2vec workflow was employed in this study. Spec2vec enhances mass spectral similarity scoring by learning structural relationships, thereby providing additional insights into the molecular networking results [31]. Inspecting the number of metabolites in both classical molecular networking and the Spec2vec network, there was minimal variation in clustering across individual settings (Figure 6). However, a notable finding was the increased inter- and intranode connectivity observed in the Spec2vec network, indicating enhanced connections among nodes within each cluster. Figure S4 highlights that acquisition settings used for Figure S2k,j detected the highest number of metabolites. Furthermore, significant improvements in the number of detected metabolites were evident when data from all settings were consolidated, as depicted in Figure S2a. The Spec2vec network facilitated putative annotations, offering comprehensive and detailed insights into the chemistry and metabolome of *Bacillus subtilis*. Analysis of the spectral network, particularly focusing on dipeptides subclusters (Figure 7), revealed distinct spectral signatures associated with phenylalanine (i.e., Phe-pro, Phe-Leu, Glu-Phe, and Asp-Phe). Moreover, as anticipated with *Bacillus subtilis*, surfactin A, B, C, and D were identified as one of the prominent metabolite classes in the bacterial extract (Figure 7). These surfactins were observed in two different clusters, highlighting their structural diversity. Within these clusters, certain nodes were not annotated via GNPS spectral library matching but are hypothesized to be structurally similar to surfactins.

**Figure 8:**
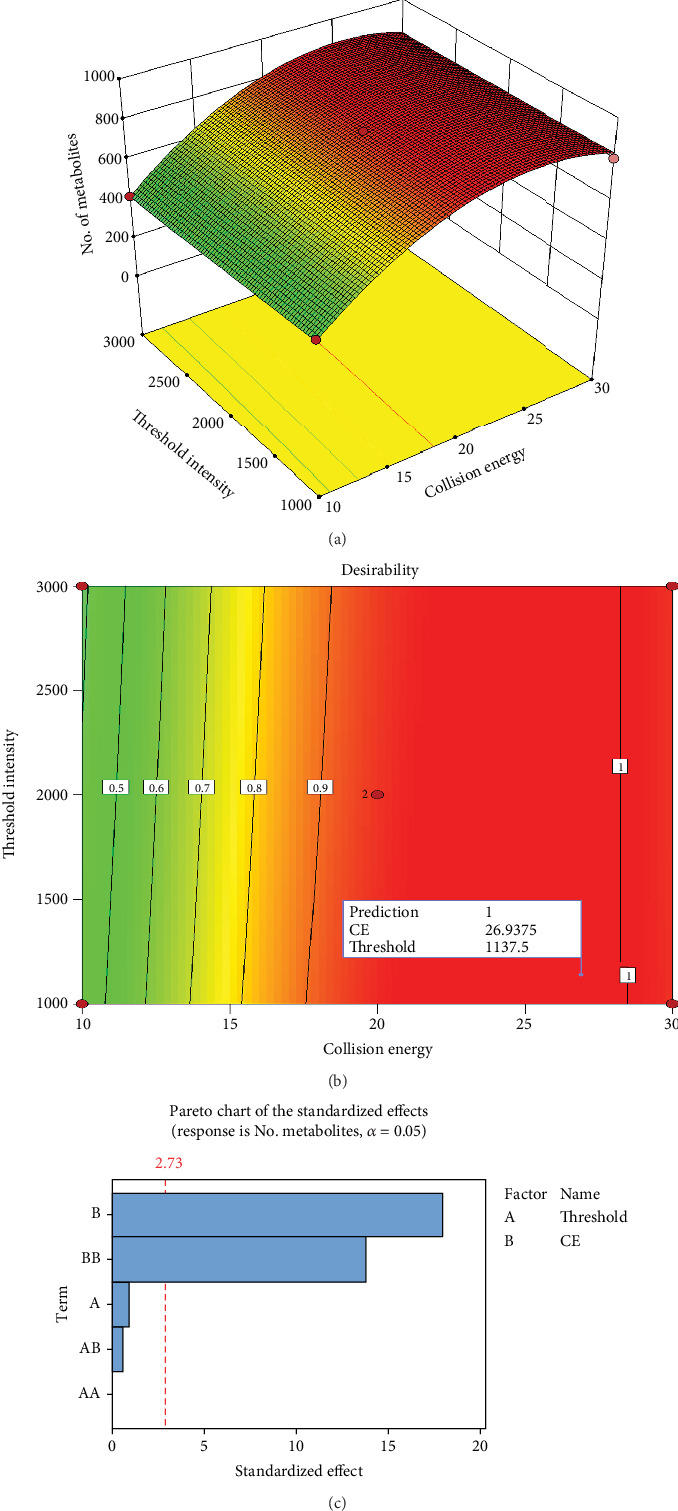
Response surfaces evaluating the synergistic effect of collision energy and threshold of intensity on the metabolite coverage. (a) 3D surface plot showing the response of number of metabolites. (b) contour plot showing the predicted optimal conditions for a wider coverage. (c) Pareto chart indicating the significant factors affecting the response.

As a result of the response surface methodology (RSM), the study established the relationship between two variables, collision energy and threshold of intensity detection, and the response value to determine the most favorable conditions for maximizing metabolite coverage. In the statistical analysis of RSM, 10 assays were conducted under varied experimental conditions. Design-Expert V8.0.6.1 software was utilized for visualization, generating 3D surface plot diagrams illustrating the interaction between the two factors based on the regression model of metabolite coverage yield from *Bacillus subtilis*. The 3D surface plots response in Figure 8 indicate that an increase in the threshold of intensity led to a decrease in metabolite coverage. Additionally, the increase of collision energy resulted in the increase of the number of metabolites detected. The observation herein aligns with the observation from the *Pareto* chart (Figure 8), indicating a significant (*p* < 0.05) linear and quadratic effect of collision energy on the number of metabolites (Table 2). In addition, the predicted optimal conditions for comprehensive metabolite coverage from *Bacillus subtilis* (Figure 8) were determined to be 26.93 eV and threshold of intensity at 1137.5, with the desirability score of 1 (as seen in Figure 8).

## 4. Discussion

The metabolite profiling of biological samples remains a global challenge; this is due to the structural diversity that these metabolites possess [32]. This diversity poses difficulties for developing innovative approaches to effectively profile metabolites within complex biological samples [33]. In general, the selection of acquisition settings in untargeted metabolomics studies may introduce biases into the data, potentially leading to redundancy and therefore compromising data robustness [34]. Considering that in metabolite profiling, the metabolites of interest are often unknown, which therefore means there is no acquisition settings that could be designed for them [35]. As such, there are no universal acquisition settings that can be applied to achieve comprehensive coverage of metabolites effectively. Hence, the present study proposes the use of multiparametric settings of the DDA approach to improve bacterial metabolite coverage through molecular networking.

In the field of metabolomics, the use of computational metabolomics tools has become a solution for analyzing the large datasets generated by advanced analytical instruments. Recently, molecular networking has emerged as a cornerstone in metabolomics data analysis; this is due to its capacity for data processing, dereplication, and visualization of chemical space [36]. In the current study, bacterial extracts were analyzed using UHPLC-qTOF-MS in positive ionization mode, and the resulting MS data were processed and interpreted using molecular networking visualized on Cytoscape. As depicted in Figures 1 and 2, the molecular networks generated show that each condition captures a distinct set of metabolites. While the number of metabolites varies significantly among the conditions, this simply indicates that each condition independently captures its own set of metabolites. As such, the results from the current study corroborate with a study by Jia et al. [37] who reported that selecting specific tandem MS settings might lead to the loss of a limited number of metabolites. This underscores the importance of careful consideration when conducting comprehensive metabolite profiling studies.

Network-based methodologies in metabolomics have garnered significant attention and have emerged as a focal point in the space of natural products [18]. However, these network-based strategies heavily rely on MS^2^ data for their analyses. In DDA, one of the key parameters that significantly influences the appearance of tandem mass spectra is the collision energy. In our study, we investigated the impact of collision energy on the generation of tandem mass spectra to achieve comprehensive coverage of the bacterial metabolome. Our results show that lower collision energy promotes fragmentation of compounds that require lower energy, resulting in some fragments having less spectral intensity. Comparatively, higher collision energy promotes the fragmentation of a larger number of metabolites, resulting in informative MS^2^ spectra, including high molecular weight molecules. Our results align with previous work, such as the study by Ramabulana et al. [24], which reported that increasing the collision energy generates a higher number of fragments in the MS^2^ spectra for a given precursor ion. However, this could be due to the structural characteristics of many natural products such as diverse bond strength, meaning that the optimal collision energy for generating informative MS^2^ spectra can vary significantly across different classes of natural products.

In comprehensive chemical analysis involving both known and unknown compounds with structural diversity, it is essential to fine-tune the threshold of intensity. In the present study, we investigated the effect of the threshold of intensity and found that a lower threshold increases the likelihood of detecting noise, while a higher threshold can filter out noise but may miss lower-abundance ions, as seen in Figure 2. This, however, influences the sensitivity and robustness of the analysis. The result from our study agrees with the result reported by Barupal et al. [38], who reported that higher thresholds can lead to the rejection of a significant proportion of low-abundance peaks. Additionally, Defossez et al. [25] highlighted that the threshold of intensity strongly depends on the signal intensity and background noise level of the mass spectrometer and that setting the threshold too high or too low can lead to limitations in metabolite coverage.

CCD is a widely used experimental design approach in optimization studies as it allows researchers to explore the effects of multiple independent variables (factors) on a dependent variable (response) efficiently. This approach has also been extensively used in metabolomics studies, from investigating the effect of ESI source parameters on metabolome coverage in untargeted LC/MS-based metabolomics [39] to optimizing protocols for processing LC-MS-based metabolomic data [40]. In the present study, CCD was used to systematically investigate which are the model or conditions that can capture the full or comprehensive metabolite coverage. Building on previous experiments that used a one-variable-at-a-time (OVAT) approach, we further investigated the effect of these two variables, since the OVAT overlooked the interaction between the two factors. Moreover, the two variables are key factors affecting the detection of compounds in mass spectrometry. The independent variables (collision energy and threshold intensity) were defined with the range from 10 to 30 eV and 1000 to 3000, respectively. In the present study, a total of 10 independent experimental setups consisting of varying settings of collision energy and threshold intensity were generated as seen in Figure 4. As evident in the vertex position of the 3D surface plot, the optimal conditions for metabolite coverage were 26.937 eV and a threshold intensity of 1100 as seen in Figure 8(b). Additionally, this further confirms the observations by Ramabulana et al. [24], which also indicated that increasing the collision energy enhances the robustness of the molecular network and expands metabolite coverage. Moreover, lower coverage of MS^2^ fragmentation was observed when high thresholds of intensity were used; this resulted in fewer nodes in the cluster. This could be attributed to the fact that lower collision energies often lead to less intense MS^2^ spectra, thereby restricting the ability of the molecular network to classify them as sufficiently fragmented for inclusion.

The GNPS-based molecular networking approach provides the opportunity to average as many MS/MS spectrum files as possible within a single spectrum group, provided that the data are of the same chemical background, thus containing similar metabolites. Interestingly, this resulted in a wider coverage of metabolites, with over 50% of the metabolites being detected. This not only improved the overall molecular network and its clustering but also enhanced the annotation of the metabolites against the GNPS library database. This improvement can be attributed to the increased number of spectra available for the algorithm to search, as having more spectral data to reference provided the algorithm with greater confidence in the metabolite identifications. Moreover, it is important to note that increasing the CE and decreasing the threshold can lead to broader coverage, which may also increase the risk of false positives. However, molecular networking makes it easier to identify such false positives because noise signal does not have a specific pattern, and such spectra typically do not match any entries in the database. Additionally, most poor-quality spectra are filtered out early in the process by excluding those with too few fragment ions. One way of experimentally standardizing this approach could be through increasing events time through data acquisition, since this is also known to increase the data points needed to generate robust data output [25]. This indicates that an increased coverage of MS^2^ acquisition, thus overcoming the limitation encountered through the semistochastic selection of abundant ion for MS^2^ fragmentation during DDA.

In our analysis, we focused on a cluster of known lipopeptides (surfactins) to examine how the improved data quality impacted the clustering within the molecular network. As shown in Figure 5, the different experimental conditions from the CCD each captured their own unique set of metabolites. Classical molecular networking revealed a broad distribution of clustered nodes across all DDA settings, with a notable concentration originating from settings characterized by high collision energy and low intensity thresholds. To complement this, Spec2vec was utilized to improve mass spectral similarity scoring through structural relationship learning, offering further insights into the molecular networking outcomes. While minimal variation in clustering across individual settings was noted between classical molecular networking and Spec2vec networks (Figure 6), Spec2vec demonstrated enhanced inter- and intranode connectivity within clusters.

The Spec2vec network facilitated comprehensive and detailed putative annotations, enhancing understanding of the chemistry and metabolome of *Bacillus subtilis*. Analysis focusing on dipeptide subclusters revealed distinct spectral signatures associated with phenylalanine derivatives (Figure 7b). Surfactins (A, B, C, and D) were identified as prominent metabolites in *Bacillus subtilis* extracts [41], observed in different clusters highlighting their structural diversity (Figure 7a,c). Furthermore, certain nodes within these clusters were speculated to share structural similarities with surfactins, despite not being annotated through GNPS spectral library matching. This suggests the presence of potentially unique metabolites, underscoring the ongoing challenge in metabolomics due to the limited spectra available in the databases for annotation. Furthermore, the annotation of the nodes was enhanced when the data from all the conditions were combined. This suggests that the molecular networking approach relies heavily on the quality of the MS^2^ data for effective clustering and visualization of related metabolites. This finding introduces a new dimension to the optimization of DDA experiments. Moreover, it raises the question of whether it is more beneficial to thoroughly optimize a single experimental condition or to systematically vary the conditions and then combine the data to achieve a more comprehensive coverage of the metabolome. By leveraging the advantages of molecular networking and the insights gained from the combined CCD data, we were able to improve the clustering and annotation of the surfactin-related metabolites. This highlights the importance of considering the overall quality and complementarity of the MS^2^ data when employing molecular networking approaches for metabolite profiling and identification.

## 5. Conclusions

In conclusion, molecular networking is the method of choice for analyzing mass spectral data in untargeted metabolomics studies due to its ability to simultaneously analyze numerous compounds. Furthermore, soil bacteria represent a promising reservoir of pharmacologically significant metabolites. However, due to the complexity and diverse chemistry of a metabolome, no single acquisition setting can effectively cover the entire metabolome. As such, the present study has demonstrated that both collision energy and threshold of intensities not only significantly influence the number of metabolites detected but also the overall data robustness and quality of acquired mass spectral data. Through CCD, the present study has demonstrated that both collision energy and threshold of intensity independently enhance metabolite coverage. However, it is noteworthy that when both parameters are combined, the comprehensiveness in maximizing metabolome coverage is even greater. Moreover, molecular networking has proven to be an effective tool for analyzing large datasets, enabling the visualization and comprehensive profiling of the metabolome. Therefore, to enhance exploration and maximize the potential of untargeted metabolomics, this study recommends transitioning from single-point optimization to group-based optimization. This approach is more viable for achieving comprehensive metabolite coverage and facilitating in-depth exploration.

## Figures and Tables

**Table 1:** Parameters that were varied for MS^2^ scan in a DDA method.

**Intensity threshold optimization experiment**	
**Intensity thresholds (counts)**	**Collision energy (eV) with a spread of 5 eV**
1000	30
2000	30
3000	30
4000	30
Collision energy optimization experiment	
1000	10
1000	20
1000	30

**Table 2:** The “model summary statistics”: focus on the model maximizing the “adjusted *R*-squared” and the “predicted *R*-squared.”

**Summary (detailed tables shown below)**					
	**Sequential**	**Lack of fit**	**Adjusted**	**Predicted**	
**Source**	**p** ** value**	**p** ** value**	**R** **-squared**	**R** **-squared**	
Linear	0.0493	0.0275	0.4559	0.1038	
2FI	0.9261	0.0249	0.3662	0.0167	
Quadratic	0.0003	0.1446	0.9842	0.9502	Suggested
Cubic	0.3768	0.1187	0.9881	0.8357	Aliased

## Data Availability

The data that supports the findings of this study are available on request from the corresponding author. The data is not available due to privacy or ethical restrictions.

## Nomenclature

ANOVA: analysis of variance
CCD: central composite design
DDA: data-dependent acquisition
DoE: design of experiments
GNPS: global natural products social molecular networking
MS: mass spectrometry
OVAT: one variable at a time
RSM: response surface methodology
UHPLC-qTOF-MS: ultra-high-performance liquid chromatography coupled with quadrupole time-of-flight mass spectrometry

## Supporting Information

Supporting Information Additional supporting information can be found online in the Supporting Information section. Figure S1: The effect of collision energy on the fragment ions. (a) Structure of surfactin C, (b) MS/MS fragmentation patterns of surfactin C at an intensity threshold of 2000 with a collision energy of 5 eV, (c) MS/MS fragmentation patterns of surfactin C at an intensity threshold of 2000 with a collision energy of 34 eV. Figure S2: Spec2vec analysis generated from ESI-positive MS/MS data, highlighting the variations in detected metabolites under different acquisition settings. (a) Combined data, (b) intensity threshold of 2000 with 5-eV collision energy, (c) intensity threshold of 3000 with 10-eV collision energy, (d) intensity threshold of 1000 with 10-eV collision energy, (e) intensity threshold of 3000 with 30-eV collision energy, (f) Intensity threshold of 2000 with 34-eV collision energy, (g) intensity threshold of 1000 with 30-eV collision energy, (h) intensity threshold of 2000 with 20-eV collision energy, (i) intensity threshold of 585 with 20-eV collision energy, (j) intensity threshold of 2000 with 20-eV collision energy, and (k) 2000 with 20-eV collision energy. Table S1: An enhanced view of the metabolome of *Bacillus subtilis* through GNPS spectra library matching and manual annotations. Spectral libraries searched includes Knacksack, Massbank, NIST, Human Metabolites, and ResSpect.
