# Supplementary material for: Multiparametric Optimization of Data-Dependent Acquisition Towards More Holistic Bacterial Metabolite Coverage Through Molecular Networking
Source: Int J Microbiol. 2025 Jul 21;2025:4388417. doi: 10.1155/ijm/4388417 (PMC12303636; doi:10.1155/ijm/4388417)
Supplement: Supporting Information — Additional supporting information can be found online in the Supporting Information section. Figure S1: The effect of collision energy on the fragment ions. (a) Structure of surfactin C, (b) MS/MS fragmentation patterns of surfactin C at an intensity threshold of 2000 with a collision energy of 5 eV, (c) MS/MS fragmentation patterns of surfactin C at an intensity threshold of 2000 with a collision energy of 34 eV. Figure S2: Spec2vec analysis generated from ESI-positive MS/MS data, highlighting the variations in detected metabolites under different acquisition settings. (a) Combined data, (b) intensity threshold of 2000 with 5-eV collision energy, (c) intensity threshold of 3000 with 10-eV collision energy, (d) intensity threshold of 1000 with 10-eV collision energy, (e) intensity threshold of 3000 with 30-eV collision energy, (f) Intensity threshold of 2000 with 34-eV collision energy, (g) intensity threshold of 1000 with 30-eV collision energy, (h) intensity threshold of 2000 with 20-eV collision energy, (i) intensity threshold of 585 with 20-eV collision energy, (j) intensity threshold of 2000 with 20-eV collision energy, and (k) 2000 with 20-eV collision energy. Table S1: An enhanced view of the metabolome of Bacillus subtilis through GNPS spectra library matching and manual annotations. Spectral libraries searched includes Knacksack, Massbank, NIST, Human Metabolites, and ResSpect. [file 4388417.f1.docx]

**Supplementary Files**


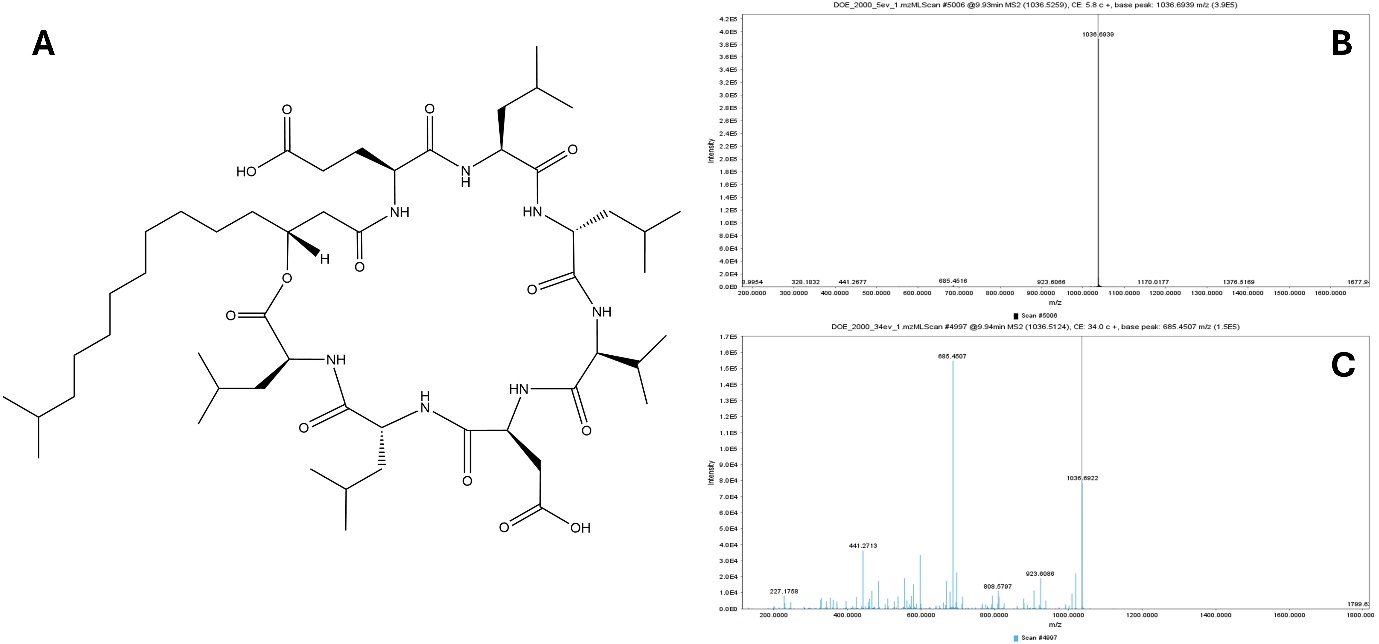


Supplementary Figure 1: Showing the effect of collision energy on the fragment ions. (A) Structure of Surfactin C, (B) MS/MS fragmentation patterns of Surfactin C at an intensity threshold of 2000 with a collision energy of 5 eV, (C) MS/MS fragmentation patterns of Surfactin C at an intensity threshold of 2000 with a collision energy of 34 eV.


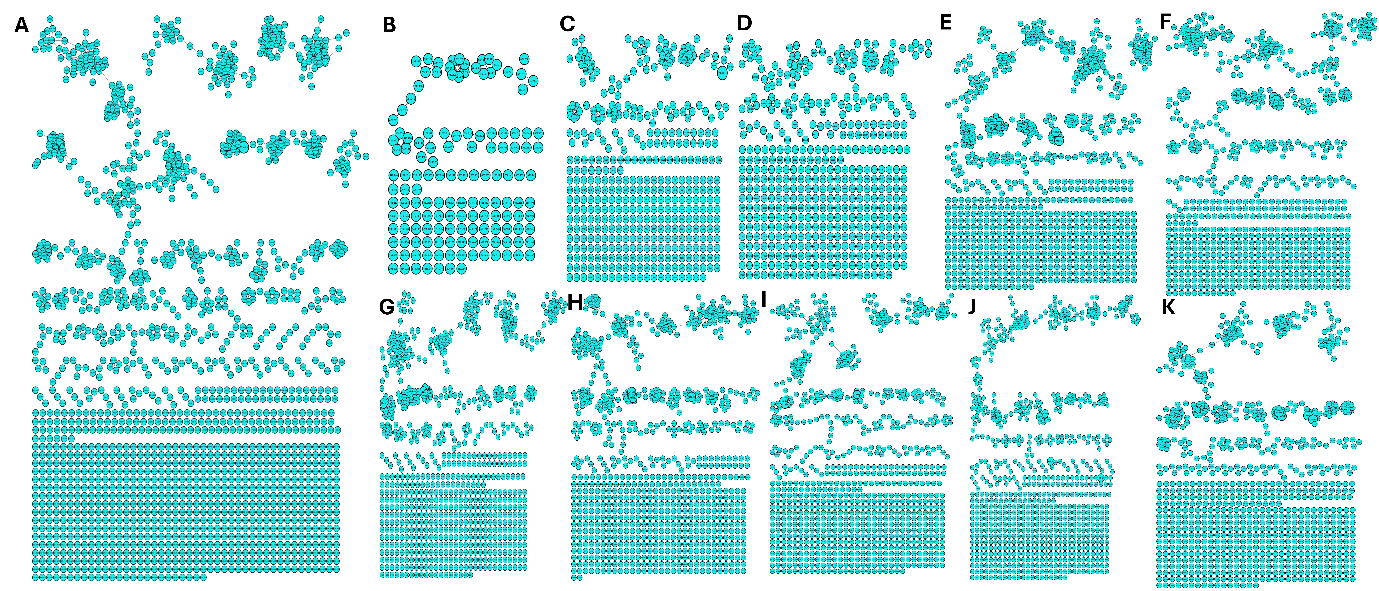


Supplementary Figure 2: Spec2vec analysis generated from ESI-positive MS/MS data, highlighting the variations in detected metabolites under different acquisition settings. (A) Combined data, (B) Intensity threshold of 2000 with 5 eV collision energy, (C) Intensity threshold of 3000 with 10 eV collision energy, (D) Intensity threshold of 1000 with 10 eV collision energy, (E) Intensity threshold of 3000 with 30 eV collision energy, (F) Intensity threshold of 2000 with 34 eV collision energy, (G) Intensity threshold of 1000 with 30 eV collision energy, (H) Intensity threshold of 2000 with 20 eV collision energy, (I) Intensity threshold of 585 with 20 eV collision energy, (J) Intensity threshold of 2000 with 20 eV collision energy and (K) 2000 with 20 eV collision energy.

**Supplementary Table 1:** An enhanced view of the metabolome of *Bacillus Subtilis* through GNPS spectra library matching and manual annotations. Spectral libraries searched includes Knacksack, Massbank, NIST, Human Metabolites and ResSpect.

| ***m/z*** | **Rt** | **Elemental composition** | **Fragment ions** | **Putative annotation** |
| --- | --- | --- | --- | --- |
| 116.0700 | 0.84 | C5H9NO2 | 116 | Proline |
| 118.0854 | 0.92 | C5H11NO2 | 118 | Glycine betaine |
| 182.0799 | 1.34 | C9H11NO3 | 165,136,119 | L-Tyrosine |
| 132.1010 | 1.60 | C6H13NO2 | 120120 | Leucine |
| 166.0853 | 2.76 | C9H11NO2 | 120 | Phenlyalanine |
| 229.1535 | 3.48 | C11H20N2O | 229, 183, 118 | Leucylproline |
| 342.2371 | 8.69 | C17H31N3O4 | 229, 183 | Isoleucyl-proline |
| 205.0959 | 5.15 | C11H12N2O2 | 188, 170 159, 146, 132 132, 118 | L-Tryptophan |
| 602.29 | 0.82 | C23H39N9O10 | 531, 389. 197 | Grgesp |
| 260.12 | 0.83 | C10H17N3O5 | 213, 156, 122, 110 | L-alanine, N[N(N-acetyl-L-alanine))] |
| 284.14 | 1.02 | C12H19N3O5 | 152, 135, 110 | L-proline, N[N(N-acetyl-L-alanine))] |
| 301.15 | 1.05 | C12H17N3O5 | 244, 155, 127 | L-proline, 1- [N(5-oxo-L-proly) glycyl] |
| 244.13 | 1.05 | C10H17N3O4 | 187, 155, 127 | Glycylprolylalanine |
| 247.13 | 1.09 | C10H18N2O5 | 229, 183, 118 | Valyl-glutamate |
| 269.16 | 1.21 | C12H20N4O3 | 223, 110 | Histidylleucine |
| 235.12 | 1.21 | C11H14N4O2 | 207, 162, 110 | Cyclo(his-pro) |
| 229.12 | 1.32 | C10H18N2O5 | 229, 183 118 | pyroglutamylvaline |
| 247.13 | 1.32 | C11H20N2O5 | 229, 183, 155, 138, 118, 102 | Glutamylvaline |
| 261.14 | 1.50 | C11H20N2O5 | 197, 148, 130, 102 | Leucyl-Glutamate |
| 432.24 | 1.51 | C19H33N3O8 | 319, 301, 206, 188, 170, 153 | ACMC-20lebal |
| 261.14 | 1.73 | C11H20N2O5 | 197, 148, 130, 102 | Leucyl-Glutamate |
| 471.22 | 1.86 | C19H30N6O8 | 357,244, 187 | Atrial peptide |
| 284.10 | 1.98 | C10H13N5O5 | 152, 135, 110 | Guanosin |
| 364.16 | 2.03 | C16H21N5O5 | 235, 209, 181, 156,152, 110 | TRH, free acid |
| 377.15 | 2.06 | C22H20N2O4 | 341, 241, 189 | Opreal-082153 |
| 251.17 | 2.14 | C12H18N4O2 | 223, 178, 110 | Cyclo(his-leu) |
| 281.11 | 2.48 | C13H16N2O5 | 234, 120, 103 | Phenylalanylasparic acid |
| 291.10 | 2.51 | C10H10N8O3 | 268, 255, 232,203,187,159 | CB Micro-033252 |
| 1030.66 | 23.3 | C51H89N7O13 | 917, 786,707,643,481,391,279,186 | Surfactin A (C13) |
| 1044.66 | 23.6 | C52H91N7O13 | 931, 800,707, 594, 481, 391,316,227 | Surfactin B (C14) |
| 1058.67 | 23.6 | C53H93N7O13 | 945, 814,707,594,463,320 | Surfactin C (C15) |
| 1022.67 | 23.3 | C52H91N7O13 | 909,794,685,582,441,338,227 | Surfactin C14 homolog |
| 1036.69 | 23.6 | C53H93N7O13 | 923,810,685,596,483,441,352,227 | Surfactin C14 |
| 239.103 | 1.9 | C11H14N2O4 | 165, 147, 136, 119 | Gly-Tyr |
| 282.279 | 0.78 | C18H35NO | 264, 195, 177, 128 | 9-Octadecenamide, (Z) |
| 366.21 | 2.63 | C17H27N5O4 | 251,234,166,156,110 | Angiotensin1/2 |
| 369.11 | 2.69 | C16H20N2O6S | 222, 204, 186, 178, 160,138 | Penicilloyl V |
| 260.16 | 3.01 | C11H21N3O4 | 242, 97,132,129 | L-isoleucine, L-glutaminyl- |
| 304.13 | 3.06 | C12H21N3O4S | 247, 155, 127 | ACMC-20mcas |
| 328.14 | 3.23 | C15H21NO7 | 310,292,264,246,178,166,143,132,120,103 | fructose-phenylalanine |
| 295.13 | 3.31 | C14H18N2O5 | 248, 120 | phenylalanylglutamate |
| 316.19 | 3.76 | C14H25N3O5 | 215,199,171,127 | threoylvalylproline |
| 227.10 | 3.90 | C10H14N2O4 | 209, 181, 139, 125 | L-proline, 5-oxo-L-prolyl |
| 325.12 | 4.31 | C19H12N6 | 296, 273, 264 | 4-(benzotriazol-1yl)-2-pridin-3-ylquinazoline |
| 302.17 | 4.73 | C13H23N3O5 | 143, 132 | Gly-Leu-Hydroxyproline |
| 223.11 | 4.75 | C11H14N2O3 | 166, 131,120,103 | Maybridge1_006768 |
| 279.13 | 4.88 | C14H18N2O4 | 232,214,158,132 | D-Proline, D-tryrosyl- |
| 188.07 | 5.15 | C11H9NO2 | 143,118,115 | indol-acrylate |
| 205.10 | 5.17 | C11H12N2O2 | 188,170,159,146,143,132,118,115,103 | Pacitron |
| 261.12 | 6.75 | C14H16N2O3 | 154, 136, 1199, 107 | Maculosin |
| 320.16 | 6.82 | C16H21N3O4 | 173, 120 | ACMC-20mguw |
| 440.25 | 6.93 | C20H33N5O6 | 229, 212 | L-proline, glycyl-L-prolyl-L-isoleucylglycyl- |
| 197.13 | 7.03 | C10H16N2O2 | 153, 133, 124 | cyclo(pro-val) |
| 555.24 | 7.06 | C24H30N1O6 | 498, 466, 247, 231, 155, 127 | opreal-_384201 |
